# In-house preparation of hydrogels for batch affinity purification of glutathione *S*-transferase tagged recombinant proteins

**DOI:** 10.1186/1472-6750-12-63

**Published:** 2012-09-18

**Authors:** Jason S Buhrman, Jamie E Rayahin, Melanie Köllmer, Richard A Gemeinhart

**Affiliations:** 1 Department of Biopharmaceutical Sciences, University of Illinois, Chicago, IL, 60612-7231, USA; 2 Department of Bioengineering, University of Illinois, Chicago, IL, 60607-7052, USA; 3 Department of Ophthalmology and Visual Science, University of Illinois, Chicago, IL, 60612-4319, USA

**Keywords:** Glutathione, PEGDA, Glutathione S-transferase, Batch purification, Recombinant protein

## Abstract

**Background:**

Many branches of biomedical research find use for pure recombinant proteins for direct application or to study other molecules and pathways. Glutathione affinity purification is commonly used to isolate and purify glutathione S-transferase (GST)-tagged fusion proteins from total cellular proteins in lysates. Although GST affinity materials are commercially available as glutathione immobilized on beaded agarose resins, few simple options for in-house production of those systems exist. Herein, we describe a novel method for the purification of GST-tagged recombinant proteins.

**Results:**

Glutathione was conjugated to low molecular weight poly(ethylene glycol) diacrylate (PEGDA) via thiol-ene “click” chemistry. With our in-house prepared PEGDA:glutathione (PEGDA:GSH) homogenates, we were able to purify a glutathione S-transferase (GST) green fluorescent protein (GFP) fusion protein (GST-GFP) from the soluble fraction of *E. coli* lysate. Further, microspheres were formed from the PEGDA:GSH hydrogels and improved protein binding to a level comparable to purchased GSH-agarose beads.

**Conclusions:**

GSH containing polymers might find use as in-house methods of protein purification. They exhibited similar ability to purify GST tagged proteins as purchased GSH agarose beads.

## Background

Recombinant DNA and protein technologies have taken a leading role in many forms of research over the past 40 years [1]. Recombinant proteins have found their way to the core of most biomedical research. Classical methods of protein purification can be divided into methods that utilize chemical properties of the protein including solubility [2], physical characteristics, e.g. isoelectric points and size [3], and those that use selective endogenous or engineered [4] protein affinity. Of this latter category, fusion proteins have emerged as a prominent method for purification. Fusion proteins are created by appending a full length or truncated protein to a terminal region of the protein of interest. For affinity fusion proteins, the appended protein will bind to a third, typically small, molecule that is immobilized on the surface of a polymer resin. The fusion protein will selectively bind to the surface while other proteins are washed away [5]. In column chromatography methods, protein lysates are eluted through columns that are packed with polymer particles. Alternatively, the affinity matrix can be mixed directly with the protein lysates, agitated for some amount of time, centrifuged and collected in batch selection methods. Fusion proteins are genetically tagged with a protein of known affinity. The glutathione S-transferase (GST)-protein is frequently used to tag a protein of interest because of its affinity for the reduced form of the tripeptide, glutathione [6]. During the elution process, excess glutathione is added to remove the tagged protein from the affinity matrix.

Traditionally, glutathione is covalently linked to the surface of activated agarose beads (Sepharose®). A thiol bond is formed between the glutathione and an alipathic spacer that is linked to a hydroxyl group on the surface of the agarose bead [7]. Since the inception of using glutathione as a capture agent for GST fusion proteins, almost all scientific supply companies carry a variation of glutathione conjugated polymeric beads, and at least one patent has been awarded [8] for its utility in proteins. Agarose has been widely used for glutathione conjugation. The chemistry of this linkage has multiple reaction and purification steps that restrict in-house production of these beads to chemistry laboratories [4,7].

Thiol-ene “click” chemistry has been shown to reproducibly form covalent thioether bonds between thiol and alkene-containing molecules. Glutathione (GSH) has been covalently linked to alkene groups in poly (N-isopropyl acrylamide) (PNIPAm) polymers [9]. While glutathione linkage to PNIPAm was confirmed, a detailed validation of the interaction with GST tagged proteins was not presented.

Using a modified form of thiol-ene chemistry and readily available materials, we demonstrate a simple, one-step method for creating gel homogenates and beads with affinity toward GST tagged proteins. Specifically, our method employs thiol-ene addition of glutathione to low molecular weight poly(ethylene glycol) diacrylate (PEGDA). Under standard conditions, the resulting polymer forms hydrogels with typical radical initiators. These gels were readily homogenized, washed, and used to purify soluble GST proteins. In our proof of concept study, GST-fused, red shifted green fluorescent protein (rsGFP) was purified with PEGDA:GSH homogenates. We also demonstrate a novel method for creating glutathione-laden PEGDA microspheres using reverse-phase emulsion polymerization. Like the homogenates, PEGDA:GSH microspheres exhibited affinity to GST-GFP and can be used to purify the protein. Either of these methods can be implemented in almost any laboratory using readily available, inexpensive reagents.

## Results and discussion

Challenging economic times combined with the influx of new scientists in biomedical research makes funding opportunities less frequent and resources increasingly strained. In response to these environmental pressures, scientists must adapt by using any available resources as tools for discovery. Newer, cheaper methods will replace traditionally more expensive techniques. Although many kits are available, most of these kits have substantial mark-up that is not always combined with quality.

To create a low-cost approach using widely available laboratory chemicals for purifying GST tagged proteins, we have utilized thiol-ene addition of reduced glutathione to low molecular weight PEGDA. We chose to look predominately at homogenization of gels as a batch purification method because most laboratories have access to plastic eppendorf homogenizers or similar. This simple method was shown to be reproducible and effective. We further develop a more complex method that has more uniformity and compares well with commercially available GST affinity beads.

### Creation of vectors & protein expression

**Figure 1:**
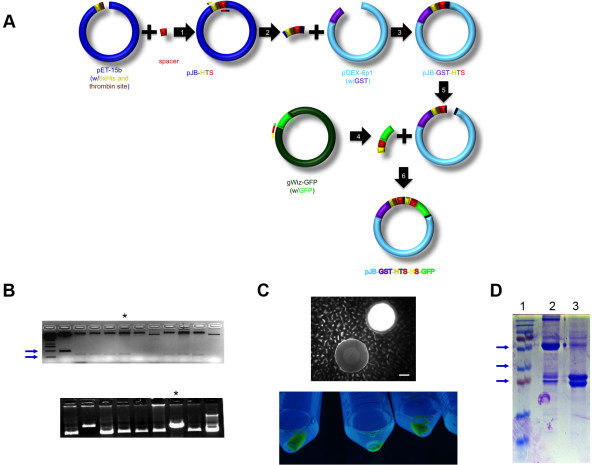
** Cloning and purification of GST-GFP. A**: Schematic of vector construction. (1) A spacer was cloned into the pET 15b vector/plasmid in proximity to the hexa-histadine tag and thrombin site, pJB-HTS (2) This fragment was cloned from the pET 15b vector (3) into the pGex-6p-1 vector to add a GST upstream, pJB-GST-HTS. (4) Red shifted GFP was isolated by primer extension with a spacer and a second hexa-histadine tag and (6) cloned into pJB-GST-HTS yielding pJB-GST-HTS-HS-GFP. **B**: Gel electrophoresis screening of DNA prepared from colonies following step 1—pJB-HTS—were digested with BglII/HindIII (top) the loss of a 500 bp band (lane 1) and appearance of a 318 bp digestion fragment indicates positive colony for spacer insertion. Arrows indicates 500 bp and 250 bp bands on marker. DNA was digested with NcoI (bottom) to screen for positive insertions. One was chosen for sequencing and further cloning (asterisk). **C**: Basal GFP expression of a pJB-GST-HTS-HS-GFP containing colony is observed microscopically (brightfield overlaid with epifluorescence) with the scale bar indicating 100 μm. After induction, bacteria express significant green color under UV illumination (bottom) indicating high levels of GFP expression. **D**: GST-GFP electrophoresed as predicted for the known molecular weight (2) before and (3) after cleavage with thrombin as compared to the (1) molecular weight ladder with arrows indicating 55 kDa, 35 kDa, 25 kDa, respectively.

**Figure 2:**
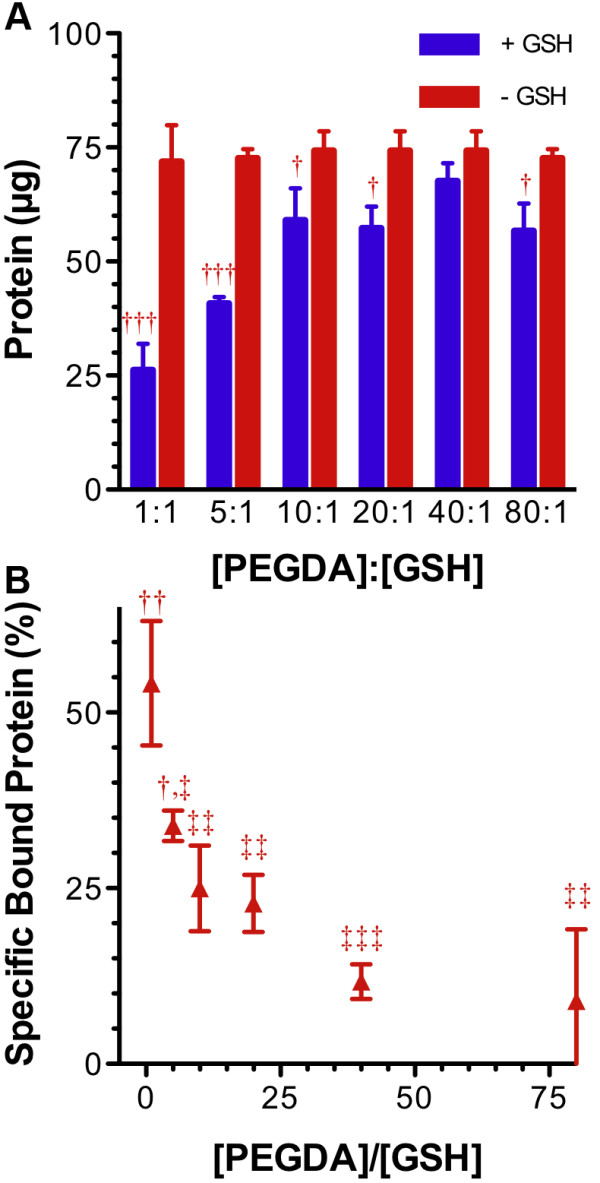
** Affinity of GST-GFP to PEGDA:GSH homogenates.A**: Influence of varying PEGDA:GSH ratio on binding of protein as expressed by the amount (μg) of protein in solution following 2 h incubation in the presence of PEGDA-GSH hydrogel homogenate (blue bars) or matched GSH-free PEGDA hydrogel (red bars). **B**: Influence of PEGDA:GSH ratio on specifically bound, or incorporated, GST-GFP as expressed as percent total GST. Total protein (100**%**) was 60 μg GST-GFP for all groups. † for p < 0.05, †† for p < 0.01, and ††† for p < 0.001. Bars and points represent the mean of 3 independents samples plus or minus (±) standard deviation.

**Figure 3:**
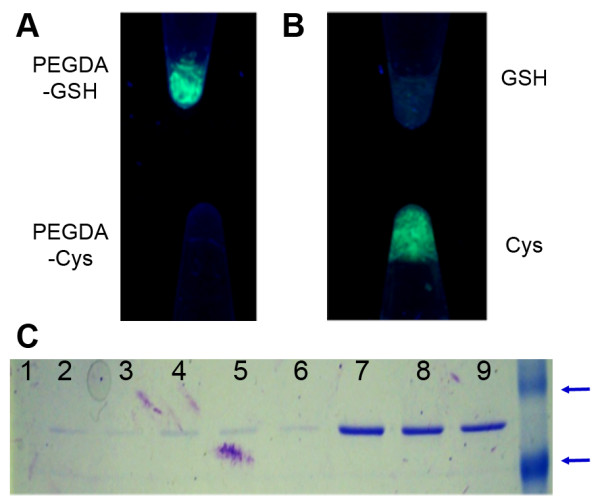
** Elution and Binding of PEGDA:GSH compared to PEGDA:Cys. A**: Gross fluorescence observation of the gels confirmed GST-GFP association with homogenates prepared with PEGDA:GSH ratio (top) or equimolar PEGDA:Cys (bottom). **B**: Gross fluorescence observation of the gels confirmed PEGDA:GSH homogenates eluted with either 10 mM cysteine (bottom) or 10 mM GSH (top). **C**: SDS PAGE electrophoresis confirmed that minimal protein eluted from (lanes 1–3) PEGDA gels eluted with GSH, (lanes 4–6) PEGDA:Cys gels eluted with GSH, but protein did elute (lanes 7–9) from PEGDA:GSH gels eluted with GSH. The last lane is a protein ladder, arrows indicating 100 kDa, and 55 kDa.

**Figure 4:**
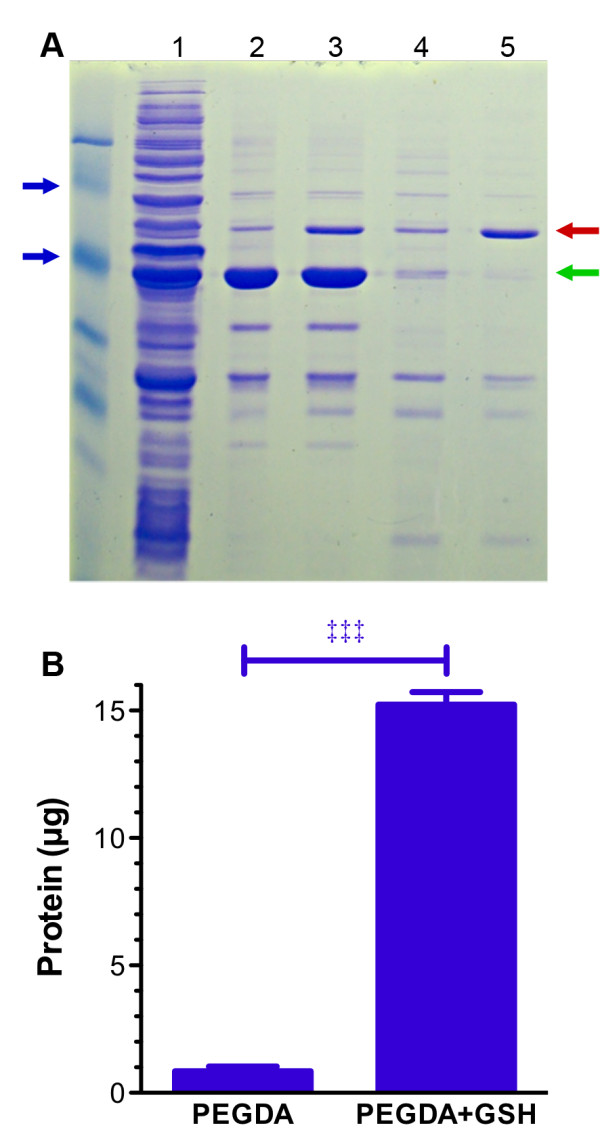
** SDS-PAGE of eluted and purified GST-GFP. A**: SDS PAGE image of proteins obtained from (lane 1) crude soluble lysate, (lane 2) after Ni purification, (lane 3) Ni purification in the presence of 10 mM glutathione, (lane 4) purification with PEGDA gel homogenate, and (lane 5) purified with PEGDA:GSH homogenate. Blue arrows indicate 100 kDa and 55 kDa while the green and red arrows are the proposed GST-GFP monomer and dimer, respectively. **B**: Protein recovered from PEGDA or PEGDA:GSH gel homogenates calibrated to total protein concentration from lanes 4 and 5 of panel. Data is presented the mean plus or minus (±) standard deviation.

**Figure 5:**
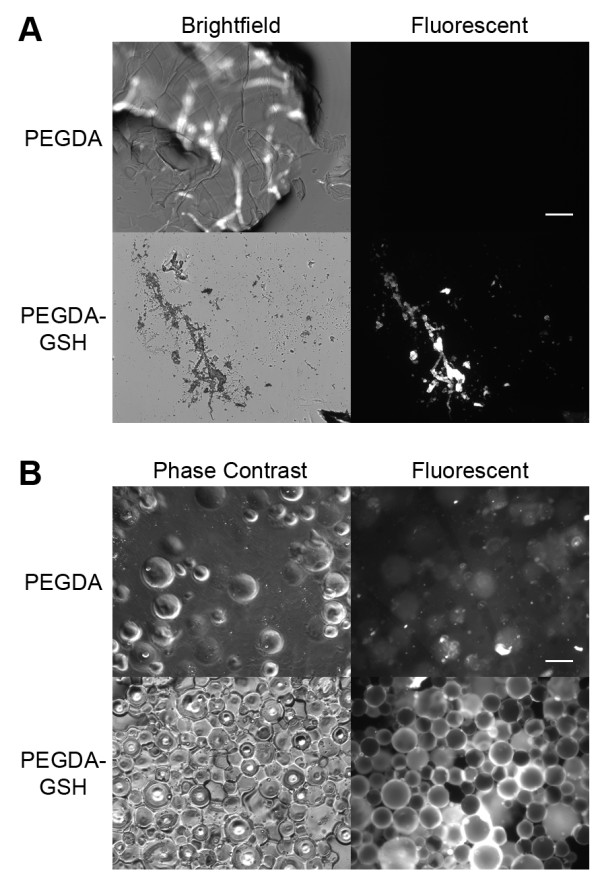
** Microscopy of homogenates and beads. Microscopy and comparison to GSH-Agarose beads. A**: Brightfield and epifluorescence microscopy of homogenates made with PEGDA (top row) or 5:1 PEGDA:GSH molar ratio (bottom row). **B.** Brightfield and fluorescence microscopy of microspheres made with PEGDA (top row) and PEGDA:GSH (bottom row) at a 5:1 molar ratio. Scale bars represent 100 μm.

**Figure 6:**
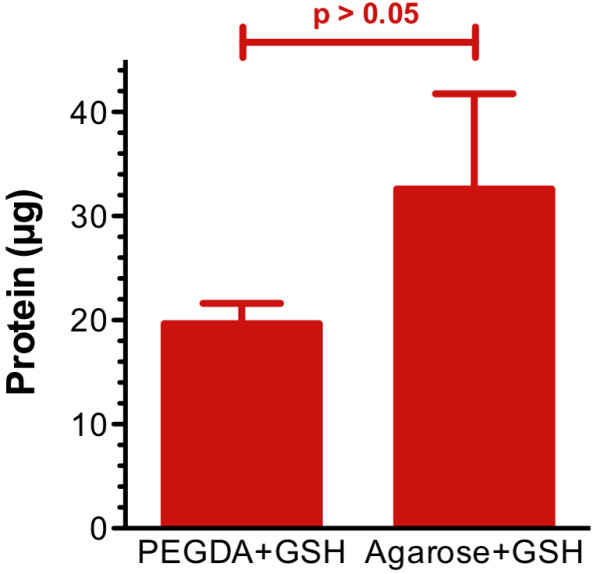
** GST-GFP association to PEGDA:GSH homogenates and PEGDA:GSH microspheres.** Protein association to GSH-agarose beads and 5:1 PEGDA:GSH microspheres. Data represent the mean plus or minus (+/−) standard error of the mean (SEM).

To create a model protein for purification, we chose to use the GFP for its solubility and ease of visualization [10]. The vector, pET 15b (Figure 1A-1), was constructed by inserting a flexible spacer sequence, a hexa-histadine tag and thrombin cleavable sequence (Figure 1B, top). The sequence was isolated by PCR (Figure 1A-2) and cloned into pGEX 6p-1 adjacent to the GST sequence (Figure 1A-3). GFP was isolated from gWIZ-GFP by PCR that added a separate spacer region and a second hexa-histadine tag (Figure 1A-4). GFP was inserted into the pGEX vector for expression (Figure 1A-5 & 6). The second hexa-histadine tag of the protein was not utilized in these experiments, but is relevant and must be noted because Ni-NTA was used to purify the GST-GFP for several of the experiments being discussed. The effect of dual hexa-histadine tags had on purification of the GST-GFP protein was not analyzed. It is clear that these dual tags did not negatively impact the protein activity (fluorescence) or binding capability to GSH. Resulting colonies were screened for gross fluorescence of the colonies (Figure 1C, top) and sequenced. GST-GFP was readily observed (Figure 1C, lower panel) and purified with a yield of approximately 300 mg/L of culture (data not shown). The nickel purified protein fraction was run on a SDS-PAGE gel to confirm size and thrombin cleavability (Figure 1D). The functionality of GST-GFP protein was confirmed by its increased affinity to nickel (data not shown) and glutathione beads, its green color and fluorescence, and its cleavability by thrombin. With our model protein produced and validated, we proceeded to validate the purification process. 

### Affinity of GST-GFP to hydrogel homogenates

Hydrogels were made as described with varying PEGDA to GSH ratios, and the gels were homogenized, washed, and incubated with 60 μg of GST-GFP for 2 h. When purified protein was incubated with the gels (Figure 2), a significant decrease in GST-GFP was found in the solution after two hours compared to gel-free controls in the 1:1 (p = 0.008), 5:1 (p = 0.005), 20:1 (p = 0.02), 40:1 (p = 0.02), and 80:1 (p = 0.02) PEGDA to glutathione ratio gels (Figure 2A). There was clear increase in bound purified GST-GFP protein (p = 0.00002; Figure 2B) when GSH was incorporated in the gels at the highest extent. The 1:1 and 5:1 ratio gels were statistically different from all other groups while the lower incorporation ratios did not associate with significantly differing amounts of GST-GFP. These experiments demonstrate significant protein association with gel homogenates harboring GSH compared to controls. As the PEGDA:GSH ratio decreased, more GST-GFP was able to associate with the homogenized gels.

After homogenization and washing, PEGDA:GSH homogenates were used to specifically purify GST tagged GFP. Further, we demonstrated that decreasing the PEGDA:GSH ratio from 80:1 to 1:1 resulted in increased GST affinity to gel homogenates. The PEGDA:GSH ratio was not further optimized because GSH inhibits polymerization reactions and gel formation at higher concentration. GSH is a known radical scavenger that decreases the number of free radicals available to sustain the polymerization cycle [11]. GSH also incorporates into acrylate groups and stops chain growth by eliminating alkene groups necessary for polymer propagation [9]. At some point the PEGDA:GSH ratio will become low enough that no usable gel will form. In this report, we focused on gels that had qualitatively acceptable and manageable physical properties, i.e. handlability for homogenization.

### Specificity of GST-GFP to GSH laden hydrogel homogenates

To confirm the specificity of the interaction, nickel-purified GST-GFP was incubated in the presences of homogenates made with equimolar GSH (Figure 3A, top) or cysteine (Figure 3A, bottom). There was no appreciable GFP observed in the cysteine-containing gels while the GSH-containing gels were visibly fluorescent. For homogenates made with glutathione, 10 mM GSH (Figure 3B, top) was able to elute the GFP completely while 10 mM cysteine (Figure 3B, bottom) was unable to elute the protein. Elution of the GST-GFP was found to be specific to GSH. No protein was eluted with GSH (Figure 3C, lanes 1–3) from hydrogels lacking GSH incorporation or eluted by cysteine (Cys; Figure 3C, lanes 4–6) from gels including glutathione. Proteins of appropriate molecular weight (Figure 3C, lanes 7–9) were eluted with GSH from GSH-containing gels.

### Purifying GST-GFP from *E Coli* protein extract

These experiments indicate that specific interactions take place between the gels and GST fusion proteins. This is not necessarily indicative of the ability of the materials to purify proteins from total bacterial lysates. Using the homogenized 5:1 PEGDA:GSH gels, crude lysates including GST-GFP from the induced, soluble protein fraction was batch selected over 2 h and eluted with 10 mM GSH. From soluble lysate, it was difficult to elucidate the GST-GFP protein (Figure 4A, lane 1). Following nickel purification (Figure 4A, lanes 2 and 3), the enriched fraction showed a high degree of purity and appropriate size as a monomer in absence of glutathione (lane 2) or as a dimer in the presence of glutathione (lane 3). Without GSH incorporation, little protein and no purification was achieved with PEGDA gels (Figure 4A, lane 4). A significant single predominant band was obtained when purification took place in the presence of PEGDA:GSH gels (Figure 4A, lane 5). The size of nickel purified GST-GFP eluted with imidazole (Figure 4A, green arrow) is half the size of GST-GFP eluted with glutathione (Figure 4A**,** red arrow). A protein of this mass is present in all samples and being twice of the size of the GST-containing monomer suggests dimerization. The absence of the GST monomer with excess GSH further suggests that the dimer would be the predominant protein present. It is known that the GST acts as a homodimer with its substrate between the two monomers [12]. From 1 mL of the initial soluble GST-GSH lysate associated with the PEGDA:GSH homogenates, 0.8 μg were eluted from the PEGDA homogenates, and 15 μg were eluted from the PEGDA:GSH homogenates. This indicates an approximate 20 fold increased affinity of the GST-GFP to 5:1 PEGDA:GSH compared to PEGDA. 

The homogenates were examined with fluorescence microscopy (Figure 5A) and it was noted that the areas of fluorescence differed in intensity inversely correlating with the thickness of the homogenized piece. Further, there was expected variation between the size of the homogenized pieces that were between 10 μm and 500 μm (data not shown). Although we felt the homogenization method may be acceptable for labs with the facilities to make the particles and that these experiments allowed us to optimize the parameters necessary for protein purification, we felt that further improvements were possible. In order to improve the yield of GSH:PEGDA purified protein and consistency, we sought more homogeneously distributed, smaller particles as we expected these to allow for more efficient association of GST-GFP to the hydrogels.

### Creation of PEGDA:GSH microspheres and establishing affinity to GST-GFP

We prepared microspheres using a reverse phase emulsion technique [13] to obtain more control over size and shape of the particles. The spheres were more homogenously distributed between 10 μm and 200 μm (Figure 5). In addition, spheres made with 5:1 PEGDA:GSH showed significant and uniform association with the GST-GFP (Figure 5B). Microspheres made from PEGDA:GSH had significantly greater affinity than PEGDA microspheres. In addition, there was no significant decrease in affinity to GST-GFP than purchased spheres made from GSH-agarose (Figure 6; P = 0.157, one tailed student’s t-test). We hypothesized that homogenous sized, spherical microspheres would increase GST-GFP association with the hydrogels by increasing the surface area of the polymer available for protein association. Microspheres were produced with PEGDA:GSH ratios as low as 5:1, but yields decreased at lower ratios (data not shown). Spheres at a 5:1 PEGDA:GSH molar ratio displayed significant interaction with GST-GFP over control gels without GSH. These easy to fabricate and inexpensive particles have great potential for protein purification. This is particularly true since the microspheres interact similarly to purchased GSH-Sepharose® beads. Homogenates were able to remove 35% of the GST-GFP from the protein solution (Figure 2B) while microspheres bind a similar amount of GST-GFP (Figure 6) at 10 fold less mass. Microspheres demonstrate an increased binding capability compared to the homogenates. This increased binding may well be attributed to an increased surface area of the microspheres compared to homogenates, but further experiments would be needed to test this hypothesis. 

Association of protein through GST-GSH interactions on the surface PEGDA microspheres introduces the exciting possibility of using these spheres for delivery of protein therapies. Building upon our previous work using proteolytically activated hydrogels [14-19], we envision using fusion proteins to release therapeutic proteins in response to disease-specific proteases. Therapeutic proteins would be released in the interstitial space [14] or intracellularly [20] by specific protease sites engineered between the GST and the therapeutic protein. Alternatively, high intracellular concentrations of reduced glutathione [21] could be used elute the GST-protein from the glutathione-containing material. Several proteases, including matrix metalloproteainse 2 (MMP-2), have been shown to be upregulated in many cancers [22,23]. Specific, short amino acid sequences have been discovered that are cleavable by MMP-2 [24-26], and could be incorporated between a tag GST and a therapeutic protein. In this way, the GST-GSH interaction could act as an anchor, holding the therapeutic protein on a hydrogel until MMP-2 cleaves the therapeutic protein from the GST anchor. Such an approach would be comparable to natural protein release from the extracellular matrix by MMPs. This biomimetic approach would advance the ability to bind protein therapeutics to hydrogels for disease-specific release. Further studies are underway to assess the potential for such a system in drug delivery.

## Conclusions

We have shown a simple and effective method for conjugating glutathione to PEGDA. These materials specifically purify GST-tagged GFP. The purity of the GFP is similar to that purified by commercial Ni-affinity agarose, and the purity and yield are compatible to purchased GST-Agarose beads. In challenging economic times we believe this simple and inexpensive method will be useful as an in-house alternative to purchasing more expensive commercial products in addition to other uses.

## Methods

### Vectors

All cloning was done in DH5alpha *E. Coli* cells and all vectors confer ampicillin resistance. The vector for protein production, pJB-GST-HTS-HS-GFP, was created by first cloning a 7 amino acid spacer fragment containing an XhoI site into pET-15b (Novagen) using sticky ligation and BamHI/XhoI sites. Colonies were mini-prepped by conventional SDS-precipitation and screened by BglII/HindIII (New England Biolabs; NEB) digestion (Figure 1B, lower panel), and confirmed by subsequent sequencing (ACGT). Primers were designed to fit in resulting vector, pJB-HTS upstream of the hexa-histadine tag (5/phos/CCATGGGCAGCAGCCATCATCAT), and downstream (AGCTGGAATTCCTAGTTATTGCTCAGCGGTGGC) (Integrated DNA technologies) of the spacer yielding a fragment containing a phosphorylated 5’ end, an initiation codon, a hexa-histadine tag, a thrombin cleavable sequence, a spacer, a termination codon, and an EcoRI site. This fragment was digested with EcoRI (NEB) and ligated into pGex-6p1 (GE Healthcare) prepared by digestion with BamHI and blunting with Mung Bean Nuclease (NEB) to generate pJB-GST-HTS. Correct insertion yields a novel NcoI site generated by ligation of the blunted ends, and so NcoI (NEB) linearization of the supercoiled vector was used to determine correct insertion (Figure 1b, lower panel). These clones were confirmed by DNA sequencing. Primers were designed to clone rsGFP from gWIZ-GFP (Aldevron) and extending a BamHI site, a hexa-histadine tag, another spacer region, the GFP, and an EcoRI site. pJB-GST-HTS and the PCR fragment were prepared by BamHI and EcoRI digestion (Fw: AAAGGATCCATCATCATCATCATCATGGTCCGCTGGGCGTTCGTGGTATGGCTAGCAAAGGAGAAGAACTC, Rev: AAAGAATTCTCAGTTGTACAGTTCATCCATGCCATG). Colonies were screened under UV microscopy for basal expression of GFP (Figure 1c). DNA was sequenced from positive colonies containing the final product, pJB-GST-HTS-HS-GFP.

### Protein expression/Purification

The vector, pJB-GST-HTS-HS-GFP was transformed into BL21 expression cells for expression and purification of GST-GFP protein. Protein was induced with 1 mM IPTG after cells reached an absorbance of 0.5 at 595 nm. They were removed to 25°C and were shaken overnight. Cells were spun at 4°C for 20 min to pellet, and resuspended in lysis buffer (50 mM NaH2PO4, 300 mM NaCl, 10 mM imidazole, pH = 8). The suspension was freeze-thawed 3x at −80°C, sonicated 3x 15 s then spun at 12 k RPM for 30 min to pellet the insoluble material. The supernatants were removed and selected through a gravity flow nickel column containing 1 mL of NTA Ni Agarose (Qiagen). After repeated washing the bound fraction was eluted with 250 mM imidazole (Fischer) in lysis buffer containing 10% glycerol (Acros).

### SDS-PAGE/Agarose gel electrophoresis

All gels in this manuscript are 15% w/v SDS PAGE gels made in-house with 37.5:1 acrylamide:bis (chemicals from sigma). Samples were prepared with Laemmeli sample buffer, and loaded into BioRad mini-protean II electrophoresis system at 150v until dye ran to end. Agarose gels were all 1.5% w/v agarose stained with ethidium bromide (Fischer) and imaged on BioRad GelDoc imager.

### Bradford assays

Bradford assay was either purchased from Pierce or made in-house as described by Bradford 1972 [27]. Absorbances were routinely read 15 min after sample addition to G-250. Homogenates were 50 mg to 100 mg wet weight. Microspheres were 5 mg to 10 mg wet weight. Each data set included BSA standards, and all protein concentrations were generated by individual BSA standard curve.

### Hydrogel homogenate production/protein binding

Hydrogels were made by adding 150 μL PEGDA (MW 575, Sigma Aldrich) to varying concentrations of glutathione (Sigma Aldrich, Alfa Aesar) and 0.05% w/v Irgacure 2959 (Ciba). PBS was used to bring total volumes to 1 mL. The tubes were cured overnight under a UV light (purchased from a local hardware store, 0.25 mW/cm^2^ 254 nm, measured on UVX radiometer). Gels were homogenized with a polypropylene EPPI-pestle homogenizer in the eppendorf tube for 10 to 20s at room temperature until most of the homogenate pieces were small enough to pass through a 1 mL micropipette tip. These homogenates were then washed with 10 mL PBS in scintillation vials (at least 5 buffer changes over two days).

### Microsphere production

Microspheres were produced by a modified reverse emulsion polymerization method [13]. Polymer solution contained 300 μL PEGDA (d = 1.1 g/mL, Sigma), 600 μL PBS, 5 μL eosin Y. Polymer solution (100μL) was transferred to glass test tube (1.5 mm diameter, 10 mm length) and 4 mL of mineral oil was added. The tube was vortexed 10s until the polymer solution formed an emulsion in the oil. Ammonium persulfate (100 μL, 20%, Sigma) was added while vortexing and continued for 1 min. Tetramethyl ethylene diamine (100 μL, 100%, TEMED, Acros) was added and vortexing continued for another minute. The tube was then left for 5 min until particles settled out, and several milliliters of deionized water were added. After most of the microspheres have settled into the aqueous phase (several minutes), oil was removed from the test tube and the solution containing the microspheres was collected in an eppendorf tube. They were then spun down, removed of their supernatant, and resuspended and washed with several changes of PBS, then washed over night in 5 mL PBS. Microspheres were compared to Glutathione Agarose purchased from Gold Biotechnology.

### Microscopy

Brightfield and fluorescence microscopy were carried out on an Olympus IX70 inverted microscope. Micrographs were captured on a Q-imaging Retiga 1300 CCD camera.

### Statistical analysis

Statistical analysis was done using one tailed students t-test for pair wise comparison or ANOVA followed by post-hoc Tukey test for multiple sample comparisons. Significance was set at α less than or equal to 0.05. Each experiment was independently repeated three times and data is presented as mean plus or minus standard deviation unless otherwise noted.

## Abbreviations

GST: Glutathione s-transferase; GSH: Reduced glutathione; GFP: Green fluorescent protein; BF: Brightfield; PEGDA: Poly(ethylene glycol) diacrylate.

## Competing interests

The authors declare that they have no competing interests.

## Authors’ contributions

RAG and JSB managed and coordinated the project. JSB, JER, MK, and RAG contributed to the design of the experiments and analysis of the data. All authors participated in the writing and editing of the manuscript. All authors read and approved the final manuscript.
